# Triads in Equine-Assisted Social Work Enhance Therapeutic Relationships with Self-Harming Adolescents

**DOI:** 10.1007/s10615-016-0613-2

**Published:** 2016-11-16

**Authors:** Catharina Carlsson

**Affiliations:** Department of Social Work, Linnaeus University, 391 82 Kalmar, Sweden

**Keywords:** Adolescents, Attachment orientations, Equine-assisted social work, Self-injury, Therapeutic relationship, Triads

## Abstract

Despite an increasing number of studies, there is still a lack of knowledge about the unique features that underlie the process in equine assisted social work (EASW). This study aimed to reveal, through qualitative methods, the dyads within the triad that become stronger during the process of EASW, as well as the effect of the participation of the horse on the relationship between the counselor and client. Data were collected through in-depth interviews with nine female self-harming clients aged 15–21 years and eight staff members. The interviews, together with video-recorded human–horse interactions with three staff members and four clients were analyzed, resulting in additional issues answered by these three staff members and four clients in a second interview. Critical dialogues between patterns and fragmentations in the narratives and video-recordings, as well as a dialogue with the participants while they were viewing videos of their own EASW sessions, led to the conclusion that adding a horse qualitatively changes therapeutic relationships in EASW. The different triads consist of different liaisons between actors in the triad, giving rise to unique combinations. The quality of the relationships depends on both the staff and the clients’ attachment orientations. Further research is needed to investigate how the degree of emotional connection to the horse affects the impact that horses have on triads in EASW.

## Introduction

Social work practices promote social change and development by building therapeutic relations and identifying needs, goals, and resources that unfold over time, representing unique combinations for every client (Adams et al. 2009). These relationships are often modeled as dyads (between two parties) and described as including trust, empathy, honesty, respect, sensitivity, responsibility, patience, active listening, the ability to negotiate, and responsiveness (Hasenfeld 2010). In equine-assisted social work (EASW), however, the therapeutic relationships are conducted with a horse, resulting in a triad. Previous studies have focused primarily on the efficacy of EASW as a method (Anestis et al. 2014) and not on the process involving the role of the third party in the therapy. Given that results in the efficacy studies have shown a discrepancy in results for the clients, interest in the process has been developed (Carlsson 2016), particularly regarding the therapeutic relationship between client and staff, which is of greatest importance in therapy or social work (Bickman et al. 2004; Duncan et al. 2003; Lundberg et al. 2015; Kim et al. 2007). Relationships with professionals are professional as well as interpersonal (Lundberg et al. 2015). Therefore, it seems important to recognize and acknowledge both the functional roles of the clients and staff that may go beyond the purview of the traditional professionalism, as well as the role of the horse. Furthermore, helpful components of this relationship are determined by individual preferences, needs and wishes. Clients and staff may perceive the horse, intervention, and triad differently (Vidrine et al. 2002). In addition, if the activity could be equally effective without the horse or if the staff interacted more with the horse than with the clients, then the therapy could not be regarded as EASW (Notgrass and Pettinelli 2014). Further, the goal in EASW is to engage in and observe the process between the client and the horse, which justifies studying the process between the client and the horse in the triad as well.

The dyadic model is limited by its disregard for the influence of one or several actors (Simmel 1971). Adding a third party not only increases the number of participants, but also qualitatively changes the relationship (Simmel 1971). A triad could consist of a liaison between two parts, but cannot be explained without considering the role of the third part. Likewise, a triad may be perceived as less threatening and engaging than a dyad, as the stigma associated with treatment clinics is removed (Brandt 2013; Richmond and Padgett 2002). However, the stable may be perceived as an intimate, protective setting for one client but as threatening or stressful for another (Bachi et al. 2012; Yorke et al. 2008).

The present study is theoretically grounded in the work of George Simmel, the forerunner of microsociology. However, the creative method (Alvesson and Kärreman 2007) selected for this study calls for a more eclectic use of theories; in this case, system theory, social psychology, and individual psychology, in the form of attachment theory. EASW relies on the therapeutic relationship in the dyad between staff and client, but could also be fueled by the client’s attachment to the therapeutic horse (Karol 2007). As highlighted by Bower and MacDonald (2001), many programs that include animals have been developed with the hope of creating an opportunity to form an attachment base with another living being. However, there is debate regarding whether EASW relates more to attachment or caregiving (Kurdek 2009). Nevertheless, Bachi (2013) argues that part of the gap between the practice and knowledge might be understood within the framework of attachment theory, the theoretical framework considered in this study.

There are indications that EASW can contribute positively to the treatment of emerging adults with psychiatric disorders. It has been demonstrated that EASW may help develop social skills and a feeling of mastery, improve meta-cognition and reflectivity, and increase self-confidence and self-esteem (Burgon 2012; Klontz et al. 2007). EASW can also contribute to the development of communication skills, enable emotional awareness and regulation, reduce anxiety, and provide the opportunity to experience authentic relationships (Bizub et al. 2003; Carlsson et al. 2014). Even though not all studies (Ewing et al. 2007; Greenwald 2001) found positive effects of EASW, it would still be useful to explore how EASW may be used in the treatment of specific client groups, such as young persons with self-injury problems.

The clients included in the present study were in residential treatment for self-injury behavior at the time of the study. Self-injury is a condition that often develops during adolescence. Known causes include a combination of an individual’s perfectionism, high standards, and low self-esteem (Holmqvist et al. 2007; Jablonska et al. 2009; Lundh and Bjärhed 2008). Emotional regulation may play a significant role in the management of the client’s condition (Gianini et al. 2013) and could be defined as an activity to regulate either the magnitude or the duration of an emotional response (Gross 2013). According to Silvers et al. (2012), emotional regulation skills in a social context are important for wellbeing in adolescence. Those adolescents who have not learned emotional self-regulation are more likely to have difficulties in school and with friendships (Silvers et al. 2012). Furthermore, attachment orientations (Zegers et al. 2006), mindfulness (Hill and Updegraff 2012) and emotional awareness (Coats and Blanchard-Fields 2008; Naim et al. 2013; Szcygiel et al. 2012) are components of EASW that seem to play a role in emotional regulation and could supplement conventional treatment.

Carlsson et al. (2014) showed that the essence of EASW is the ability of the horse to open the client to greater emotional awareness and regulation, facilitating a relationship between the client and the staff member that is perceived as more authentic. Horses are sensitive animals, which require human body language to act according to the emotions felt (Chamove et al. 2002; Minero and Canali 2008). A person’s attitude toward the horse often directly affects the behavior of the horse (Hama et al. 1996). All humans, whether staff members or clients, therefore need to regulate their emotions in the presence of a horse. However, previous studies have not explored the role of the horse for the staff members. The horse responds to human emotions through body language, which is perceived as immediate, honest, clear, and nonjudgmental (Carlsson et al. 2014). The horse offers projection opportunities, which the staff member and client can explore in “real time.” This “real time” projection is unique to therapy of this type (Bachi et al. 2012). However, this projection could also have potentially negative effects on the secure base of the client, according to Bachi (2013); the horse walking away from the client may be perceived by the client as a signal of dislike. Thus, the intervention of the therapist is crucial in interpreting the projection (Bachi 2013). The essence of EASW is enhanced by staff members when they focus on the client’s emotions and help the client to understand that the horse is acting based on the client’s and staff member’s behavior, at the same time as they regard the horse as a subject (Carlsson et al. 2015).

Findings from an earlier study (Carlsson et al. 2014) indicated that one outcome of EASW could be a decreased resistance to change. If the horse did not elicit clients’ defense mechanisms, clients might be more willing to go beyond their comfort zones. Thus, the horse seemed to set the framework for the interactions between staff and clients (Carlsson et al. 2014). However, clients’ age, aversion to horses, experience with horses, motivation, severity of problems, and length of time spent with horses as well as interference from others in the setting could influence how the triad is perceived (Hauge et al. 2013; Schultz et al. 2007).

Therefore, the purpose of this study was to elucidate whether the triad would induce a qualitative change in how the therapeutic relationship between client and professional is perceived. The research questions were as follows:

1. How should the third party (the horse) in the triads in EASW be considered? Do participants in triads form different dyads within the triads, in which one dyad is more indirect? Specifically, is the horse consistently a tertiary member of the triad?
2. Knowing the relationship between staff and clients is of importance, how should we consider the horse’s contribution to the dyad between the counselor and the client? Are participants’ mutual relations affected by being part of a triad?

## Method

### Participants

The treatment center was chosen because it provided individual treatment with horses for clients that was separate from usual treatment, enabling the specific observation of EASW. The director of the treatment center was contacted and informed about the study. Permission to conduct the study was then granted by the director. All staff and clients at the center were given information about the study in both written and verbal form. Participants were informed that the video-recorded material would be used solely for the purpose of the study. Both clients and staff signed an informed consent form in accordance with ethical guidelines. All 12 enrolled clients at the treatment center were female self-harming adolescents, aged 14–21 years. The clients came from different regions in Sweden, and all but one had Swedish ethnicity. All 12 clients at the treatment center were asked to participate; two had not commenced treatment with horses when the study started and therefore did not attend. One of the clients had not reached 15 years of age and was excluded because of difficulty obtaining consent from her parents. Finally, nine clients participated in the first interviews of whom four were selected for the observations. These four clients were selected because they had had participated in EASW for more than one semester and, therefore, were considered to be less affected by the observation and also able to provide more detailed descriptions of the treatment compared to those who had just begun. Two other clients out of those nine with similar durations of treatment, and who had the most experience with horses among all clients who participated in EASW were not observed because they dropped out of the treatment. Clients and staff who were observed participated in a second interview with questions based on an analysis of the first interviews.

Additionally, eight staff members who worked with horses as a complementary treatment or activity were interviewed. All staff members had previous experience with horses and all owned at least one horse. Staff members came from various educational backgrounds, namely, social pedagogy, social work, psychotherapy, nursing, and riding in combination with assisting treatment. Only those who worked in the complementary EASW treatment were observed. In addition all staff who were observed had undergone a 3-year riding-therapy educational program. One other staff member who met the inclusion criteria was excluded due to not having an EASW client at the time of the study. Although the staff came from various educational backgrounds, all had specific education and experience in cognitive behavioral therapy (CBT) and dialectic behavioral therapy (DBT), which formed the theoretical basis for their work with the clients.

The horses that were observed varied in terms of breed, age, gender, experience, temperament, and size. The breeds included in this study consisted of Shetland ponies, Icelandic horses, Lusitanos, Dutch warmbloods, and Norwegian fjord horses. In total six different horses were included in the study but only four were observed, when the girls worked with one specific horse during the sessions. The treatment center allowed the horses plenty of free movement in a herd, which enabled social contact with other horses. These factors have been found to be important to ensure a calm and safe environment for the participants (Hartmann et al. 2012).

### EASW Intervention

This study was conducted at a Home Care and Housing Treatment Center, in a facility with a riding arena, stables, and therapy room. The area around the facility, such as woodlands and meadows, were also used. EASW was offered for 1 h weekly and used as a complement to the regular program (CBT). The primary emphasis in EASW was not therapeutic riding, unlike with the physically disabled. Rather tasks ranging in difficulty, such as returning the horse from the field, grooming (brushing the horse, plaiting the mane), riding (in the arena or for a walk in the forest), or stable work was employed. Being skilled in the activities was not the focus. Clients kept the same horse as much as possible throughout the intervention. Participants were not required to have previous experience with animals or horses before participation. Several clients were initially frightened, but eventually appreciated the horses. Clients continuously learned about the horses to interact safely with them. The main goal of EASW is to work hands-on with clients’ day-to-day issues and to bring attention to their interactions with horses. Clients were helped to develop awareness of their thoughts and emotions and to increase their ability to regulate their affect. This therapy is also thought to motivate clients to develop attachment behaviors and skills (Johansen et al. 2014). The EASW treatment was tailored for individual clients based on their treatment goals and desires. It was conducted individually due to clients’ need for perfection and the previous discomfort previously experienced when working in groups. Staff also set boundaries regarding what could be done in each session based on the safety of the client and the horse. The temperament, behavior, and willingness to interact of each horse were assessed daily. Client assessments included a mental status examination. If a client exhibited a negative emotional status (e.g., signs of aggression), the session with the horse was cancelled for safety reasons; however, such instances were rare. In addition, staff seldom ended sessions abruptly; premature ending of a session was usually due to the client’s empathy for the horse. Clients knew that the horse would not like emotions such as fear or anger and therefore asked staff to support them with another exercise not involving the horse.

### Procedure and Analysis

For the first 40–60 min, in-depth interviews based on six to seven themes (based on a narrative review of previous research published by Author 2016) were conducted separately with the clients and the staff at the treatment setting, except for one interview that was conducted with a staff member at a hotel. Conducting interviews in the same manner with both staff and clients enabled comparisons between these groups. Next, video-recorded observations were conducted with each triad of staff, client, and horse working together in EASW over three separate 1-h sessions each week. Pilot observations were conducted in two separate sessions with each triad before video recording to enable habituation to the researcher’s presence. To develop trust prior to the observations, the researcher, who was not known by the participants, also visited several times. To facilitate the analysis, observations were video recorded for the full length of the EASW session (Heath et al. 2010), which provided an opportunity to observe the participant’s emotions, expressed verbally or non-verbally. A mobile video camera and familiarity with the activities (via supervising practitioners and lecturing students in EASW) facilitated the video recordings (Heath et al. 2010). In direct conjunction with the third video-recorded observation (thus better enabling informants to provide rich descriptions of their experience), the clients and the staff members were interviewed separately with open questions based on an initial narrative analysis (Barkhuizen 2014) of the first interviews. All interviews and observations, as well as field notes made after the interviews and observations, were recorded and transcribed verbatim.

When the observer could record only one of the participants, the choice was always to follow the client. The video camera was held about 2–5 m from the participants when they were grooming and working close to the horse in the stable, and about 5–10 m when riding or driving in the paddock or outdoors. Observations were video-recorded to verify whether clients and staff were not aware of certain aspects of the interaction and therefore did not mention them. Video recordings also made it possible to explore different issues on different occasions (Heath et al. 2010) without participants having to undergo extensive observation. The videotape also provided opportunities for other scientists to observe and review the analysis. Additionally, participants had the opportunity to comment on their experiences (Heath et al. 2010); in the final interview, participants were asked to make comments while examining the video. These interviews were utilized as analytical resources, not just as descriptors, according to the creative method (Alvesson and Kärreman 2012).

The interview transcripts and videotaped observations were analyzed through phenomenological analysis. The contents were grouped by clusters of meaning, supported by the data program MAXQDA. This process involved multiple viewings, coding of categories, identifying relationships, and comparing of categories until central themes emerged. The process used both inductive reasoning through constant comparison and deductive reasoning to generate variables from an existing theory. The empirical data were compared between clients, between staff, and between clients and staff to search for patterns across cases (Smith et al. 2012). Inspired by ethology using animal behavior methodology, communication between the client and horse, as well as between the staff and horse were studied. Records of behaviors such as looking at the horse, making contact with the horse, fear of the horse and degree of leadership over the horse, were made. Contact initiated by the participants toward the horse, as well as toward each other was recorded as physical, verbal, or visual (eye contact). Noted responses of the horse included the position of the head and ears, as well as if the lips were raised, showing the teeth. Following the categories used by Hauge et al. (2013), ears pricked forward and/or turning the head in the direction of the participant were regarded as positive responses, ears relaxed to the side and head lowered were regarded as neutral responses, and ears pinned backwards and/or head turned toward the participant while raising the lips and showing the teeth were regarded as a negative responses. Horses’ responses were observed to determine if horses could be perceived by participants as attachment figures or as transitional objects according to attachment theory. Horses might perceive frustration or anxiety in humans and respond negatively (Russell 2003). Happiness, on the other hand, might be regarded as neutral by horses and calmness as positive. Thus, horses’ responses could reflect feelings of the participants (Carlsson et al. 2014), enabling the inference of participants’ emotional status even when not verbalized. Field notes by the researcher accompanied by constant dialogue with reference persons—students, practitioners, and researchers in the field of human–horse interaction—were made to evaluate the validity and reliability of the study.

## Results

**Fig. 1:**
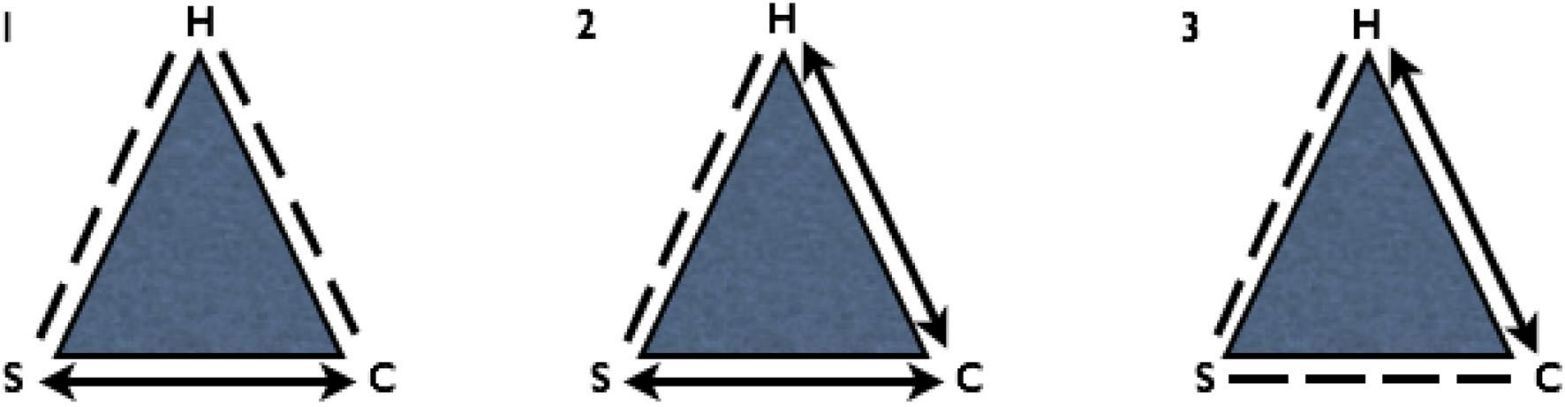
Three types of triads. *H* horse, *S* staff, *C* client. *Dotted lines* are intended to indicate varying intensity in the relationship, to signal a more indirect relationship between the participants at the ends of the *dotted lines*

Based on the analysis, three types of triads emerged (Fig. 1):

1. Triads based on trust in the dyad between the staff member and client—indirect relation to the horse;
2. Triads based on trust in double dyads between the staff member and client as well as between the client and horse; and,
3. Triads based on trust in the dyad between the client and horse—necessary to form a relationship between the staff member and client

### Trust in the Staff-Client Relationship—Indirect Relationship with the Horse (1)

The dyadic relationship between the client and staff member forms the base of the triad; this was observed in two client–staff relationships in this study. In this triad, the third agent, the horse, has a more indirect relationship to both the staff member and client. Thus, the relationship between the client and staff member is not dependent on the horse. According to one of the two clients, staff members were always available despite strain in the therapeutic relationship. Thus the linking function was mainly reliance on staff due to their knowledge about and experience with horses. Both of the staff members in these triads had a long history with horses, and their experience with animals was for them a substitute for family and friends during various periods in their lives.

I have lived in the countryside and been slightly disconnected from access to peers. Then the horses became a stand-in. Absolute best friend, competition buddy, and the therapist with whom I have cried and laughed (Staff L).

This regard for horses did not seem to be transferred to clients in the triads, for whom the horse was regarded more as an object. In such triads, the relationship between the client and staff member formed the foundation; clients distinguished between relationships with animals and relationships with humans.

But she (Staff H) knows a lot about me. She knows me well, and I think it will be harder to say goodbye to her than it will be to say goodbye to the horse, because that is a totally different relationship. /…/ I mean between a human and an animal there is one kind of relationship and between a human and a human it is a much more dynamic and irreplaceable relationship/…/ it can rarely be exactly replaced. Animals though, they can be replaced (Client H).

Although the horse had an indirect role in this triad, it nevertheless played a role. The horse was not perceived by clients as a being with whom to form an emotional attachment; rather, the horse was perceived as that which could be blamed if the task at hand was not achieved. Thereby, the horse could reduce problems in the relationship between the staff and client.

We have good moments in everyday life, but it becomes pretty useful to bring in a third agent, someone you can blame for things going wrong or someone you need to relate to and explore together (Staff L).

In these triads, the horse was perceived more as an object. This might mean that the staff and clients, although having some feelings for the horse, were more task-oriented. They may respond to the needs of the horse, but were more concerned with achieving the goal rather than responding to nonverbal cues from the horse. The setting also seemed to be perceived by staff as protective when they stated that the work in the triad together with the horse reduced staff members’ performance anxiety.

Even if the clients are freaking out, as long as I have control over the horse, I do not get stressed. /…/ The considerations for the horse are our first priority because we cannot use the horse over its limits. That makes me confident in setting boundaries for the clients based on what the horse is capable of (Staff D).

Again the horse could be perceived as reducing problems in the dyad by taking the blame for restraining the actions of the clients.

### Trust in the Relationship between Staff and Client and between Client and Horse (2)

This triad is based on two dyads consisting of the relationship between the client and staff member as well as the relationship between the client and horse. This does not mean that the staff member has no relationship with the horse. On the contrary, the staff member has extensive experience with horses, considers them to be friends and working partners, and promotes the importance of having a close relationship with the horse in therapy. The staff member in this triad preferably works with her own horses, although that was not the case in this study. The staff member in this triad reported that she was not confident that the horse acted as well as a co-therapist as her own horse would have done. The staff member did not have an emotional connection to the horse in the study and thus, she thought she could not respond as well to the nonverbal cues from the horse. Even though the staff member could not read the horse in the study as well as her own in all situations, the horse could still have had an impact on the situation.

I think I am actually much more calm and stable as a person or therapist in cooperation with a horse than I am as a private person or as a treatment assistant (Staff L).

In this triad, both the staff member and client had a history with different animals in their lives. They shared earlier positive experiences with horses with each other. The staff member added that the experience of seeing what horses could do for humans motivated her to conduct such work with clients. The participants in this triad agreed that in the beginning of their lives, horses filled their need for activity and could be perceived as a stand-in for peers. They both experienced close friendships with horses they previously owned. The attachment between staff member and client seemed to strengthen after sharing common experiences with the horse.

Further, in this triad, it was apparent that the client was very fond of her therapy horse. The client felt an emotional connection to the horse and was interested in responding to nonverbal cues from the horse. The horse could initiate or end the interaction and the client was perceived by the staff member and herself as having an attachment to the horse. The client mentioned that she wanted to buy the horse and bring her back home when the treatment was completed:

I want to show the director of the treatment center that I really enjoy being with E (the horse) and show her (the director) what I can accomplish, so it could be possible for me to bring her (the horse) home, finally. So she can become mine/…/ I could not say goodbye to her, she means too much to me/…/that would probably be the worst thing that could happen to me (Client G).

The client reported recognizing herself in the horse: both were stubborn and sensitive, according to the client. The connection between them was also rooted in the client’s perception that the therapy horse always paid attention and listened to her. This experience was not as frequent with staff members in general.

The horse listens but does not answer the same way as a human and it does not get angry in the same way. / … / It is nice that it does not say anything back (Client G).

Additionally, the staff member and horse shared similar characteristics: they were both kind, stubborn, and mischievous, according to the client. Client G added that, although it was not as easy to hug the staff member as it was to hug the horse, it was still easier to hug staff member L than other staff members.

It’s pretty easy to hug staff member L. … I like to hug staff member L, she is kind. She usually always asks if I want a goodnight hug and I like that (Client G).

The relationship between the staff member and client was described, by the client, as having an additional dimension in EASW than therapy in the treatment center. It seemed like the emotional connection between the client and staff member was stronger in EASW. In the treatment center, staff was described as more task-oriented and preoccupied with clients achieving goals in school and so on. Staff was more stressed with daily work at the treatment center according to both client and staff.

When we are doing therapy with the horse, we (staff member and client) are best buddies. She (the staff member) helps me so very much and knows just how I am and all that stuff. Like, for example, in the treatment center on an ordinary day, she (the staff member) can be quite tedious (Client G).

### Trust between Client and Horse Necessary to Form a Relation between Staff and Client (3)

In this triad, the horse has a linking function; without the horse, the same connection would not have occurred between the client and the staff member. The staff member said that she connected in a completely different way with Client A in EASW compared to in the treatment center.

I see and understand the girls better with the help of the horse /…/ I have met some girls where I have changed from hardly being able to be in the same room with them to feeling really lucky to be in the same room, and that happened in just a couple of weeks (Staff G).

It seems like the staff in this case had difficulties bonding emotionally with clients at the treatment center. Staff members responded to clients’ needs and were concerned about tasks and achievement when working in the treatment center. In the stable, this seemed to change to a response to nonverbal cues rather than a response to needs. The client also spoke about a better understanding with staff and realizing that the staff member was interested in what she does, thinks, and feels:

She [the staff member] is like a completely different person now since I started having her in equine-assisted social work. Before, Staff G was just the angry boss and I hated her because she would just make decisions about everything. Then I thought, “f**k.” She can talk about her problems/…/ She told me about her feelings and how sad she was about having to remove one of her own horses. Suddenly, she became a person to me, not just the boss. Then, I started to like hanging out with her. Before, she was the angry boss. Now, she is more a person as well. Yes, I like her a lot more now than I did before (Client G).

Nevertheless, the horse was the important character in the triad according to the client; a friend whom the client longed for when no one else was around. This was because the horse was perceived as more honest, which was something Client G could not always say about staff members. Moments with the horse were pure happiness, according to Client G, and she felt that the horse loved her, something she hardly ever felt with humans. Consequently, Client G, as well as the staff, learned the importance of respecting each other from the horse. After the client showed that she could respond to the horse’s nonverbal cues and respected that the horse could want to end or initiate the interaction, the staff member’s impression of the client changed. The staff member perceived Client G as more empathetic, which strengthened the relationship between the staff member and client. Finally, both Client G and the staff member learned that first impressions should not determine a relationship. The client’s approach to horses also changed along the way, from treating the horse like an object, to treating it as a being (subject) with a personality. Client G reported that she now realized it was much more about trying to understand what the horse was feeling and whether the horse was doing well, something that is also true for people even if they do not always say what they mean and want.

## Discussion

Almost as many types of triads as there were clients were found in this study. In one triad, the staff member was most important to the client and the horse was replaceable, if mistakes could be attributed to the replacement. However, if the horse could be replaced, then it would technically not be EASW, according to Notgrass and Pettinelli (2014). The opposite was true of the last triad, for which the horse was the foundation for the triad and responsible for building a bridge between the staff member and client. The results also showed a triad in which the horse and the staff member were equally important to the client. In this triad, the client seemed to have developed an attachment to both the staff member and the horse. When humans have an emotional bond with an animal, social support is perceived as stronger, according to Antonacopoulos and Pychyl (2008). This bond may be created through patting and grooming the animal, as seen in this study and an earlier one (Bachi et al. 2012). When social support is related to self-esteem, this type of triad could be argued to have a greater influence on self-esteem due to emotional bonds existing with both the staff member and the horse. However, earlier studies have not differentiated between types of triads; previous studies instead reported no impact on self-esteem (Ewing et al. 2007; Holmes et al. 2011; Kaiser et al. 2006) or an impact on self-esteem (Burgon 2012; Klontz et al. 2007) after interventions with horses. Our results on different triads indicate that further research is needed on self-esteem in relation to different triads in social work with horses. Varying results regarding self-esteem in previous research could be related to self-harming adolescents have higher levels of self-criticism related to self-esteem (Jablonska et al. 2009; Lundh and Bjärhed 2008).

In this study, three different types of triads were found to be qualitatively different regarding attachment. In the first triad, the focus seemed to be more on caring for the horse rather than attachment, consistent with Kurdek (2009). On the other hand, in the last triad, it was the attachment to the horse that caused the staff member and client to reach out to one other, in line with Bachi (2013). Karol (2007) also argued that the intervention with the horse not only relied on the therapeutic relationship with the staff member, but was also fueled by the client’s attachment to the horse, as seen in the second triad. Studies have also shown that clients often comment on their connection to the horse and how much they enjoyed being together with “their” horse (Maujean et al. 2013). In addition, Bachi (2013) reported that many aspects of attachment theory—safe haven, affect mirroring, reflective functioning, and nonverbal-communication— applied not only to the clients but also the staff members. Inclusion of animals can provide a save haven and secure base for staff when engaging in challenging and complex processes with clients (Zilcha-Mano et al. 2011), which was seen in the present study. Regarding the last triad, the staff’s ability and willingness to provide a safe haven seemed to be dependent on the relationship between the client and the horse. Therefore, it would be important to explore further the staff members in these settings; earlier research indicated that an insecure or avoidant staff member was less likely to empathize accurately or lacked the skills needed to provide sensitive care clients (Mikulincer and Shaver 2007). Further, as secure attachment can only occur when a person is calm and focused (Mikulincer et al. 2012), there is reason to examine narratives of both the staff and clients regarding whether the horse had a calming effect on them during EASW.

Early patterns of attachment shape, but do not determine, expectations in later relationships, according to attachment theory (Bowlby 1979). As Bowlby (1988) later claimed, attachment is important throughout life and is manifested in thoughts, emotions, and behaviors related to support seeking. In addition, previous research (Edenburg 1995) has shown that humans may have different internal working models of their attachment to people versus their attachment to animals. Earlier research (Carlsson et al. 2015) has shown that EASW is facilitated when staff help clients understand their feelings and actions as well as understand others’ reactions to these actions by mentalizing, in other words, by being good attachment figures. As Bachi (2013) argues, equine-assisted work can provide physical and emotional safe havens as well as provide a secure base from which clients can explore and learn to develop their own capacities and personality. If the client–staff relationship or the client–horse relationship can become a safe haven and secure base for the client, then it can facilitate healthy emotion regulation and the exploration of new possibilities, which contribute to mental health (Mikulincer et al. 2012). Furthermore, both clients’ attachment insecurities as well as staff members’ own sense of security could facilitate or prevent positive therapeutic processes and outcomes (Mikulincer et al. 2012), making further exploration of the triads identified in this study of interest. In particular, research could focus on whether staff should work with their own horses when they have indicated that their attachments to their own horses are different. Siporin (2012) argued, however, that the therapy horse is more than a large teddy bear or transitional object. The horse is a sensitive animal; thus, its concerns and emotions must be attuned to in the presence of humans (Siporin 2012). Regardless of whether social support comes from the horse, the setting, or the staff member, it is important to study further the relationships between participants in EASW. This is highlighted by the findings of a previous study that showed adolescents with the lowest level of social support before participation in interventions with horses displayed the greatest improvement in handling the horse during the intervention (Hauge et al. 2014) or the greatest percentage improvement in scores after treatment (Trotter et al. 2008).

Moreover, the present study demonstrates that understanding therapeutic relationships is not just a question of adding up the various dyadic constellations. In a triad two parties always relate to the third one, regardless of whether the latter is actively present or has a passive role in the background, in accordance with Simmel’s (1971) theories; thus the therapeutic relationship in EASW seems to change qualitatively when adding a third party. The different triads consist of different liaisons between actors in the triad. However, even though the client has the closest relationship with the staff member, this does not mean that we should not consider the client’s relationship with the horse. The bond between the client and staff member could bring out the usefulness of the horse in the triad. Similar results could be seen when the client has the strongest bond with the horse. In these cases, other qualities in the staff member became visible, resulting in a qualitatively different relationship between the staff member and client. The thoughts, feelings, and behaviors of the client seemed to be affected by the presence of the third party, even when the third party was present in different ways in the triad, in accordance with social psychology theories (Fiske 2004). As earlier research has shown, the building of therapeutic relations in social work could result in unique combinations between clients and staff members (Adams et al. 2009). In earlier studies about triads, Khurana (2002) differentiated between three types of triadic constellations, in which one consists of a member (the client, in this study) benefitting from the relationships in the triad. Although the examples given by Khurana (2002) were not entirely similar to those in the current study, they demonstrate the complexity of triads. Triads involve tension between proximity and distance, a central analytical theme for Simmel (1971). Using the bridge as a metaphor to illustrate distance and proximity, the bridge connects but, at the same time, also marks a detachment (Kaern 1994); the horse could be seen as a bridge builder, but it could also be said that a gap exists in the relationship between the client and staff member. The same could be said when the staff is the bridge builder: then, the gap is highlighted between the client and horse. This phenomenon raises questions about who is there for whom in therapeutic relationships in EASW, and whether the horse is there primarily to meet the need of the client or those of the staff member. Following Hasenfeld (2010), who argues that relationships in social work are supposed to include trust, empathy, honesty, respect, sensitivity, responsibility, patience, active listening, the ability to negotiate, and responsiveness, then, there is reason to question if this always held true in this study. As an example, clients highlighted that they were not accustomed to trusting or respecting staff members. More research is therefore needed to determine why the trust for the staff members occurred in EASW. Further, most previous studies have been concerned with outcomes in EASW, and a discrepancy has been identified between qualitative and quantitative studies, such that qualitative research tends to demonstrate greater effects (Pauw 2000). The results of this study highlight the importance of closely examining the effect on staff as well as the effect on the staff-client relationships regarding trust, empathy, etc. (Hasenfeld 2010). One solution could be to distinguish between statistically significant effects and clinically meaningful effects (Bachi 2014; Pauw 2000).

The present study has limitations, however. We are unable to draw conclusions about the long-term effects of EASW triads. Rather, this study suggests that girls in the process of EASW demonstrate abilities matching those that were improved in previous studies, such as feeling of mastery, communication skills, emotional awareness and regulation, self-confidence and self-esteem, and reduced anxiety (Pendry and Roeter 2013; Rothe et al. 2005; Smith-Osborne and Selby 2010). Another issue of concern is that, for example, that a person’s self-image can be diverse, fragmented, contradictory and dependent upon the contexts (Searcy 2007). However, this study did not examine clients’ self-images or self-esteem in depth. Thus, researchers should further investigate these topics and how clients could regulate their emotions within triads in EASW, when self-esteem, self-image, and emotional regulation play a significant role in the maintenance of their conditions (Gianini et al. 2013). Further, research should also consider how the emotional connection between staff members and the horse affects these triads in EASW: does the degree of emotional connection affect the impact the horse has in the triad, as some of the staff argued? Do the staff members’ perspectives of the client change depending on the emotional connection to the horse? Moreover, this study has limitations concerning generalizability, or transferability. The small sample does not allow us to create an index of transferability. However, the rich descriptions allow the reader to make informed judgments about the transferability of the findings to other settings. Nevertheless, the results correspond with earlier research, with practical experiences of referees who work with similar and different client groups, as well as with students’ experiences of acting as clients with horses undergoing training in EASW, indicating transferability. Using different sources of information (staff members and clients), different methods, as well as different theories arguably increased the validity of the study.

## Conclusion

In the field of equine-assisted interventions, research has mainly focused on identifying evidence for the method studied even when it is not concluded that interventions assisted with horses as EASW could be regarded as a method of its own (Carlsson 2016). Further, while methods and manuals alone do not ensure positive outcomes among users of services, future research should investigate the development of standardized and manual-based methods. It could be argued that focus on methods and manuals on the other hand neglects the large amount of evidence of the importance of the relationship between the professional and client (Lundberg et al. 2015) and impedes understanding of the helpful components within the complexity of services available. The present results indicate that staff members sometimes are purely designated as professionals, while in other cases they are regarded as a people. The helpful components of the relationship are connected more directly to one or the other dimension; however, neither in isolation is sufficient to understand the helpful aspects of the relationships (Lundberg et al. 2015). Processes within these relationships, whether with staff or horse, as well as clients’ individual preferences, needs, preconditions and opinions are central to understanding EASW, as being open to the fact that every relationship is unique and that what is helpful can vary. The concept of a neutral and distant staff may seem limiting when that seem to limit the closeness between client and staff member; however, there is no evidence that the relationship to the horse is automatically closer.
